# Back-up Arteriovenous Fistulas in Peritoneal Dialysis Patients: A Systematic Review and Meta-analysis

**DOI:** 10.1016/j.xkme.2024.100904

**Published:** 2024-09-19

**Authors:** Hicham I. Cheikh Hassan, Pauline Byrne, Christie Harrod, Donia George, Karumathil Murali, Jenny H.C. Chen, Judy Mullan

**Affiliations:** 1 School of Medicine, Lebanese American University, Beirut, Lebanon; 2 Department of Renal Medicine, Wollongong Hospital, Wollongong, NSW, Australia; 3 Graduate School of Medicine, Faculty of Science, Medicine and Health, University of Wollongong, Wollongong, NSW, Australia

**Keywords:** Back-up arteriovenous fistula, dialysis access, meta-analysis, peritoneal dialysis, systematic review

## Abstract

**Rationale & Objective:**

Peritoneal dialysis (PD) is a dialysis modality limited by the potential need of transferring to hemodialysis. Optimal hemodialysis vascular access is an arteriovenous fistula. Back-up arteriovenous fistula (bAVF) is a strategy to prevent central venous catheter (CVC) insertion, but its use in the PD population has not been systematically reviewed.

**Study Design:**

Systematic review and meta-analysis.

**Setting & Study Populations:**

Studies including PD patients with a bAVF and the associated outcomes, including risk of hemodialysis transfer with a CVC and the proportion of bAVFs used.

**Selection Criteria for Studies:**

Retrospective or prospective, observational studies, non-randomized or randomized controlled trials.

**Data Extractions:**

Vascular access at time of hemodialysis transfer (bAVF vs CVC) for patients with and without a bAVF. The data on bAVF outcomes included bAVFs that stopped working, were never used, and the number of patients requiring hemodialysis.

**Analytical Approach:**

Random-effects meta-analysis and meta-proportional analysis were conducted, with risk of bias within studies assessed using the Newcastle-Ottawa Scale.

**Results:**

We screened 1,855 studies, 11 of which met the inclusion criteria, comprising 598 (62%) patients with a bAVF and 368 (38%) without. The proportion of bAVFs never used was 69% (95% confidence intervals [CI], 0.58-0.80; *I*^2^ = 86.2%). Meta-analysis of 8 studies found no difference in hemodialysis transfer between patients with a bAVF and those without (hazard ratio, 1.14; 95% CI, 0.86-1.51). However, the risk of hemodialysis transfer with a CVC was significantly lower in patients with a bAVF (hazard ratio, 0.43; 95% CI, 0.17-0.68).

**Limitations:**

Substantial heterogeneity between the studies and large number of studies with poor quality.

**Conclusions:**

bAVF was associated with a high rate of non-utilization but a lower risk of starting hemodialysis via a CVC. Future studies assessing long-term clinical outcomes may provide further insights into the role of bAVF creation in shaping dialysis unit policies.

## Plain-Language Summary

Peritoneal dialysis (PD) is limited by hemodialysis transfer. The optimal vascular access in hemodialysis is the arteriovenous fistula, yet requirements for maturation often necessitate a central venous catheter (CVC) insertion in acute transfers from PD. A back-up arteriovenous fistula (bAVF) is a strategy used to avoid CVC use in such situations. However, no consensus is currently available on the best approach for bAVF in PD. By conducting a systematic review, we found that 69% of bAVFs were never used. Nevertheless, bAVFs reduced the risk of hemodialysis transfer with a CVC by almost 60%. The studies in the review were small in number and exhibited high heterogeneity, emphasizing that further research is needed to clarify the role and benefits of a bAVF in PD patients.

Peritoneal dialysis (PD) is one of the preferred treatment options for patients with kidney failure. PD allows for preservation of residual kidney function,^1^^,^^2^ flexibility of treatment schedule, maintenance of quality of life, reduced health care costs,^3^ and equal, if not better, mortality outcomes compared to in-center hemodialysis (HD).^4^^,^^5^ Despite these advantages, one of the main barriers for successful long-term PD management is transfer to HD, which occurs in 40%-50% of patients.^6^^,^^7^ Within the first year alone, almost 20% of PD patients in Australia, New Zealand, and the United States transferred to HD.^8^^,^^9^ The most common cause of transfer to HD is infection, with the peritoneum being the leading site of infection.^6^, ^7^, ^8^

The ideal vascular access for HD is an arteriovenous fistula (AVF) highlighted by the “fistula first approach.”^10^ HD patients starting with an AVF have a lower risk of mortality and bloodstream infections and achieve better blood flow rates than patients starting with a central venous catheter (CVC).^10^ However, AVFs require planning, preparation, and time to allow for maturation before use. In PD patients, an AVF may not be ready for use when transfer to HD is abrupt, especially with an acute infectious episode such as peritonitis. An observational study in the United States estimated that up to 80% of patients starting HD from PD within the first year of PD initiation did so with a CVC.^9^

Consideration of PD may be hindered by concerns of transferring to HD shortly after PD initiation with a CVC, especially in high-risk patients. An attempt to provide reassurance has led to some centers adopting a plan to create a back-up AVF (bAVF). Studies of the use and outcome of bAVFs span the last 40 years.^11^^,^^12^ However, this practice has never been systematically studied. To address this gap in evidence, we aimed to systematically review the utility and outcomes of bAVFs in patients receiving PD.

## Methods

This systematic review and meta-analysis was conducted according to a prespecified protocol and in accordance with the Meta-analysis Of Observational Studies in Epidemiology^13^ and the Preferred Reporting Items for Systematic Reviews and Meta-Analysis guideline for conducting a meta-analysis.

### Search Criteria, Data Sources, and Search Strategy

A systematic literature review was conducted to identify studies examining the use and outcomes of bAVFs in the PD population as follows: *Population*: Adult patients commencing or receiving PD treatment. *Intervention*: Creation of a bAVF, defined as an AVF created in anticipation of HD before or after PD start, or as defined by the authors. *Comparators*: PD patients with no bAVF or without comparator. *Outcome:* Primary outcomes were transfer to HD and dialysis access at time of HD transfer (bAVF vs CVC). Secondary outcomes were complications of bAVFs (proportion of functioning bAVFs and utility of bAVFs). Dialysis access at HD transfer was defined as the vascular access used in the first HD session. *Methodology:* Full-text or abstract publications of retrospective or prospective studies, observational studies, or non-randomized (without controls) or randomized controlled trials.

We searched MEDLINE, Embase, and Cochrane Central Registry of Controlled Trials (CENTRAL), from inception until May 31, 2022 using a combination of keywords, Medical Subject Heading terms, and subject headings for bAVF or AVF and PD. The search strategy is provided in Item S1. We also examined the reference lists of articles to identify other studies. References were exported and managed with Endnote X20 software (Clarivate Analytics).

### Study Selection and Eligibility Criteria

Titles and abstracts in the initial search were screened independently by 3 authors (PB, CH, and DG) based on the eligibility criteria. Eligible studies were included for independent full-text review by the same authors. All discrepancies were resolved by consensus.

### Data Extraction, Study Verification, and Quality Assessment

For publications meeting the selection criteria, data were extracted independently by 3 authors (PB, CH and DG) using a standardized data extraction form and checked by another author (HCH). Discrepancies were resolved through consensus. The following data were extracted: study characteristics (journal, author, year of publication, publication type [article or abstract], country, study design, duration of follow-up), age, sex, total number of patients, number of patients with a bAVF and with no bAVF. Outcome data included the number of bAVFs that stopped working or were never used and the number of patients with bAVFs who required HD (total number, using a bAVF, or using a CVC). We defined bAVFs that were never used as those that stopped working or were never used because of death, transplantation, or remaining on PD. For the meta-analysis, the outcome was comparing the bAVF and non-bAVF groups for the risk of HD transfer and the risk of starting HD with a CVC.

Risk of bias assessment was conducted by 3 reviewers (PB, CH, and DG) independently, and disagreements were resolved by consensus. We used the Newcastle-Ottawa Scale for observational studies^14^ used in the meta-analysis. Newcastle-Ottawa Scale scores ≥7 were considered high quality, 5 to 6 moderate quality, and <5 low quality.

### Statistical Analysis

We conducted meta-proportional analysis and meta-analysis. The meta-proportional analysis examined outcomes specifically in the bAVF group. Tests of significance on the pooled proportions was calculated using the double arcsine transformation to stabilize the variance as suggested by Freeman and Tukey.^15^ A sensitivity analysis was conducted excluding studies published before 2000, given the large change of practice between the older and more recent publications. For the meta-analysis, we used a dichotomous outcome with pooled risk ratios and 95% confidence intervals (CIs). Because of the heterogenous nature of the included groups, pooled estimates were obtained using the random-effects model, and heterogeneity was examined using forest plots and *I*^2^ statistics, with *I*^2^ values of 25%, 50%, and 75% corresponding to low, moderate, and high levels of heterogeneity, respectively. Publication bias was evaluated by funnel plots if >8 studies were included. Statistical analysis and generation of forest plots was conducted using Review Manager (Rev Man) 5.4 software (Cochrane Collaboration), with *P* < 0.05 deemed statistically significant. For the meta-proportional analysis, the *metaprop* program in STATA was used.^16^

## Results

### Study Selection and Characteristics

**Figure 1:**
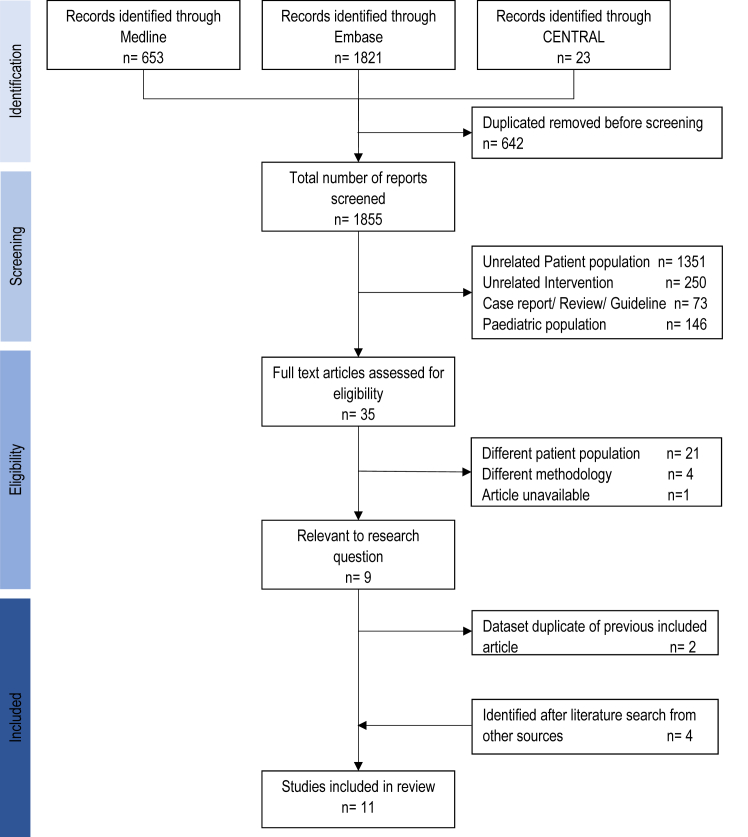
Flowchart of study selection in the meta-analysis. CENTRAL, Cochrane Central Register of Controlled Trials.

We identified 1,855 abstracts and retrieved 35 full-text articles (Fig 1). We included 7 articles published between 1986 and 2021 for analysis after excluding 2 studies that had the same patient population. Four studies were included after examining the reference lists of included studies. Our final analysis included 11 studies,^11^^,^^12^^,^^17^, ^18^, ^19^, ^20^, ^21^, ^22^, ^23^, ^24^, ^25^ all retrospective cohort studies, for the meta-proportional analysis while 8 studies had a non-bAVF comparator group and were suitable for the meta-analysis.^11^^,^^12^^,^^17^, ^18^, ^19^^,^^23^, ^24^, ^25^

General characteristics of the included studies are presented in Table 1.^11^^,^^12^^,^^17^, ^18^, ^19^, ^20^, ^21^, ^22^, ^23^, ^24^, ^25^ The studies included a total of 1,332 patients, and baseline characteristics were available and accessible for 598 patients with a bAVF and 368 with no bAVF. The smallest study had 12 patients with a bAVF^24^ while the largest had 115 patients.^12^ The mean age ranged from 48-62 years.

**Table 1:** General Characteristics of Included Studies

Author (Year)	Country (Number of Centers)	Journal (Publication Type)	Start to End Date Follow-up Time	Study Design	Patient Number	Males n (%)	Age (y)	Follow-up (mo)	Cohort Characteristics
Joffe et al^11^ (1986)	Denmark (Single center)	*Perit Dial Int* (Article)	Nov 1979-Jul 1985 69 mo	Retrospective parallel	43	28 (65%)	57.3 (NA)	16.8 (NA) Range 1-62	Cause of KF: GN 35%, chronic pyelonephritis 16%, diabetes 11%, HTN 9%, PCKD 9%, chronic interstitial nephritis 5%
Farrington et al^18^ (1991)	United Kingdom (Single center)	*Nephron* (Letter)	Jan 1986-Dec 1987 24 mo	Retrospective parallel	76	41 (54%)	50.7 (13.7)^a^ 49.6 (20.0)^b^	19.8 (9.7)^a^ 17.1 (10.6)^b^	Diabetes 13%
Divino Filho et al^17^ (1991)	Brazil (Single center)	*Nephrology* (Article)	Jan 1984-Jun 1988 54 mo	Retrospective parallel	66	NA	NA	NA	NA
Beckingham et al^19^ (1993)	United Kingdom (Single center)	*Lancet* (Article)	Jan 1986-Dec 1989 48 mo	Retrospective parallel	176	NA	NA	48 (NA)	NA
Chui et al^20^ (2000)	Australia (Single center)	*Hong Kong Med J* (Article)	Jul 1986- May 1994 95 mo	Retrospective single	136	82 (60%)	49 (NA) Range 13-75	26.4 (NA) Range 0.7-92.2	Cause of KF: GN 24%, chronic pyelonephritis 8%, diabetes 11%, HTN 2%, analgesia 16%, renovascular 3%, other 24%
Jiang et al^21^ (2013)	Australia (Single center)	*Nephrology* (Article)	Jan 2006-Jun 2010 69 mo	Retrospective single	35	18 (56%)	71 (62,79)	11.5 (6.1, 21.9)	Diabetes 44%, HTN 84%, PVD 22%, CAD 50%, cerebrovascular disease 19%
Nezakatgoo et al^22^ (2017)	United States (Single center)	*Perit Dial Int* (Short report)	Jan 2012-Sept 2015 45 mo	Retrospective single	24	10 (42%)	50.7 (13.2)	19.6 (NA)	Diabetes 46%, CAD 33%
Ferreira et al^23^ (2018)	Portugal (Single center)	*Perit Dial Int* (Article)	Jan 2014-Dec 2016 36 mo	Retrospective parallel	183	103 (56%)	55.2 (14.8)	42.1 (25.6)	Diabetes 31.3%. Cause of KF: GN 16%, diabetes 28%, HTN 17%, PCKD 7%, interstitial nephritis 3%, other 30%
Ng et al^24^ (2018)	Australia (Single center)	*Nephrology* (Abstract)	Jan 2017-Apr 2018 16 mo	Retrospective parallel	43	27 (63%)	62 (NA) Range 19-86	NA	NA
Haralabopoulos et al^25^ (2020)	Australia (Single center)	*J Nephrol* (Article)	Jan 2006-May 2018 149 mo	Retrospective parallel	176	108 (61%)	61.7 (49.2,71.6)	NA	Cause of KF: diabetes 35%, other 65%.
Rao et al^12^ (2021)	Australia (2 centers)	*Int Med J* (Article)	Jan 2009-Dec 2013 60 mo	Retrospective parallel	374	214 (57%)	65 (50, 75)^c^ 57 (42, 60)^d^	NA	Diabetes 25%, CAD 28%, PVD 22%.

Notes: Data expressed as mean (SD) or median (IQR) unless specified otherwise.

Abbreviations: bAVF, back-up arteriovenous fistula; CAD, coronary artery disease; GN, glomerulonephritis; HTN, hypertension; IQR, interquartile range; KF, kidney failure; NA, not available; PCKD, polycystic kidney disease; PD, peritoneal dialysis; PVD, peripheral vascular disease; SD, standard deviation.

a Group A: Patients with a bAVF and a Tenkoff catheter for PD.

b Group B: Patients with a Tenkoff catheter only for PD.

c Center where all patients received bAVF if feasible.

d Center where only patients considered high risk of PD failure received bAVF.

Publication bias was not assessed because <8 studies were included in the meta-analysis. Risk of bias was assessed in the 8 observational studies containing a control group using the Newcastle-Ottawa Scale (Table 2).^11^^,^^12^^,^^17^, ^18^, ^19^^,^^23^, ^24^, ^25^ Five studies had a score of 7 or 8, indicating good quality, while 3 studies had a score of ≤4, indicating poor quality. The lower scores were due to lack of comparability of the patient groups and the lack of the outcome of interest (AVF) at the start of the study.

**Table 2:** Risk of Bias Assessment (Based on Newcastle-Ottawa Scale)

Author (Year)	Selection ∗∗∗∗	Comparability ∗∗	Outcome ∗∗∗	Sum (Maximum 9)
Joffe et al^11^ (1986)	∗∗--	-∗	--∗	4
Farrington et al^18^ (1991)	∗∗∗∗	∗-	∗-∗	7
Divino Filho et al^17^ (1991)	-∗--	-∗	---	2
Beckingham et al^19^ (1993)	∗∗∗∗	-∗	∗-∗	7
Ferreira et al^23^ (2018)	∗∗∗-	-∗	∗∗∗	7
Ng et al^24^ (2018)	∗∗--	--	---	2
Haralabopoulos et al^25^ (2020)	∗∗∗-	-∗	∗∗∗	8
Rao et al^12^ (2021)	∗-∗∗	∗∗	∗-∗	7

Notes: Selection criteria (4 stars): Representation of exposed cohort, selection of non-exposed group, ascertainment of exposure, demonstration that outcome of interest was not present at start of study. Comparability (2 stars): Comparability of cohorts by the design or analysis. Outcomes (3 stars): Assessment of outcomes, follow-up duration, and adequacy of follow-up of cohorts.

### Meta-proportional Analysis of bAVF Outcomes in PD Patients

**Figure 2:**
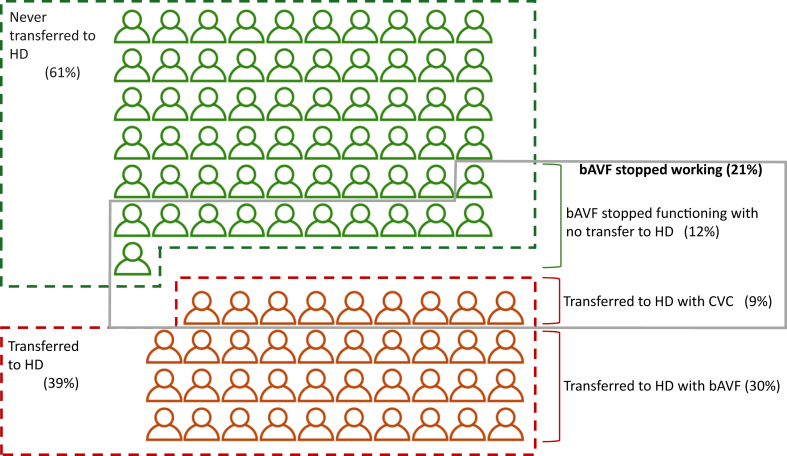
Representation of the outcome of 100 patients with a bAVF based on the meta-proportional analysis. In the figure, green depicts patients who never transferred to HD (due to death, kidney transplantation or those who remained on PD) and red patients who transferred to HD. Abbreviations: bAVF, back-up arteriovenous fistula; CVC, central venous catheter; HD, hemodialysis; PD, peritoneal dialysis.

The characteristics of the bAVF group, along with the outcomes of the included studies, are summarized in Table 3.^11^^,^^12^^,^^17^, ^18^, ^19^, ^20^, ^21^, ^22^, ^23^, ^24^, ^25^ The bAVF outcomes of interest included the proportion of bAVFs that stopped functioning or were never used, as well as the proportion of bAVF patients who started HD (bAVF or a CVC). Not all of these outcomes were examined in each study. The proportion of patients whose bAVFs were never used (defined as never used due to death, transplantation, patient remained on PD modality, and bAVF that stopped working) was 0.69 (95% CI, 0.58-0.80; *I*^2^ = 86.2%) (Table 4). This included patients who never transferred to HD due to kidney transplantation (0.08; 95% CI, 0.01-0.20; *I*^2^ = 84.2%), death (0.11; 95% CI, 0.03-0.23; *I*^2^ = 76.7%), and patients who remained on PD modality (0.25; 95% CI, 0.10-0.44; *I*^2^ = 87.7%). In the entire cohort, the proportion of patients whose bAVF stopped working was 0.21 (95% CI, 0.13-0.31; *I*^2^ = 70.9%). The proportion of patients with a bAVF who started HD was 0.39 (95% CI, 0.30-0.49; *I*^2^ = 79.9%), with most patients commencing HD through the bAVF (proportion, 0.30; 95% CI, 0.20-0.42; *I*^2^ = 87.1%) and a minority with a CVC (proportion, 0.08; 95% CI, 0.04-0.15; *I*^2^ = 77.5%). These findings are visually depicted in the forest plots of the proportion of outcomes (Figs S1-S8). Significant heterogeneity was present between studies for the outcome assessing bAVF never used, needing HD, and needing HD by CVC or bAVF (*I*^2^ > 75%), while heterogeneity was moderate for the outcome assessing bAVF not working (*I*^2^ = 71%). Figure 2 shows the proportion of patients who never transferred to HD, those who transferred to HD, and those whose bAVF stopped working. A sensitivity analysis was conducted excluding studies published before 2000 (Table 4). The estimates of outcome, 95% CI, and heterogeneity were similar.

**Table 3:** Outcomes of Included Studies With Information on bAVF Cohorts

Author (Year)	bAVF (n)	bAVF Site	Definition of bAVF Group	Stopped Working, n(%)	Never Used, n(%)	Required HD, n(%) Total; bAVF; Line	bAVF Procedure	Reason for HD Transfer
Joffe et al^11^ (1986)	19	NA	12 with AVF from HD transfer 7 bAVF created before PD start	2 (11%)	15 (79%)	4 (21%); 2 (11%); 2 (11%)	NA	Peritonitis and inadequate dialysis
Farrington et al^18^ (1991)	56	NA	bAVF concurrent with PD catheter insertion (Incomplete data on reasons for bAVF creation.)	19 (34%)	39 (70%) Tx 14 Death 12 RPD 5	25 (47%); 17 (30%); 8 (14%)	2.73 (1.1)^a^	NA
Divino Filho et al^17^ (1991)	23	NA	16 with AVF from HD transfer 7 bAVF created before PD start	6 (26%)	11 (48%) Tx 0 Death 0 RPD 11	12 (52%); 12 (52%); 0	NA	Peritonitis, inflow/outflow obstruction, exit site infection, hernia
Beckingham et al^19^ (1993)	114	Mainly radiocephalic, occasional brachiocephalic	bAVF created before PD start as back-up unless not surgically suitable	NA	103 (90%)	33 (29%); 10 (9%); 2 (2%)	1.34^b^	Peritonitis (80%), perforation (6%), acute posttransplant use (14%)
Chui et al^20^ (2000)	33	NA	bAVF concurrent with PD catheter insertion	NA	16 (49%)	NA; 17 (52%); NA	NA	NA
Jiang et al^21^ (2013)	35	All in upper arm	bAVF concurrent with PD catheter insertion in patients deemed high risk of long-term PD failure	9 (26%)	25 (71%) Tx 0 Death 10 RPD 9	16 (46%); 10 (22%); 6 (17%)	NA	Peritonitis (63%)
Nezakatgoo et al^22^ (2017)	24	Brachiobasilic 33%, brachiocephalic 33%, radiocephalic 21%, radiobasilic 13%	bAVF concurrent with PD catheter insertion in patients with no functioning AVF or multiple failed attempts, life expectancy >12 mo and not for live transplant	8 (33%)	20 (83%) Tx 2 Death 1 RPD 12	9 (38%); 4 (17%); 5 (21%)	33% pre-HD procedure	Peritonitis (33%), pleuroperitonal fistula (11%), inadequate PD (44%)
Ferreira et al^23^ (2018)	85	Distal 47%, proximal 46%, unknown 6%, shunt 1%	40% bAVF created before PD start 60% bAVF created during PD in anticipation of HD	NA	51 (60%)	36 (42%); 34 (40%); 2 (2%)	NA	Peritonitis 42%, ultrafiltration failure 29%, mechanical 13%, others 17%
Ng et al^24^ (2018)	12	NA	5 AVF concurrent with PD catheter	4 (33%)	7 (58%)	5 (42%); 5 (42%); 0	NA	Transplant, peritonitis, inadequate PD, abdominal surgery
Haralabopoulos et al^25^ (2020)	82	NA	bAVF created before PD start or up to 6 mo after PD start if patient considered risk of failing PD	13 (16%)	40 (49%) Tx 14 Death 9 RPD 8	51 (62%); 42 (51%); 9 (11%)	NA	Peritonitis (25%), dialysis inadequacy (26%), patient preference (10%), abdominal hernia (6%), catheter issues (2%), dialysis leak (2%)
Rao et al^12^ (2021)	115	Radiocephalic 76%	bAVF created in all patients unless not suitable	10 (9%)	93 (81%)	25 (22%); 22 (19%); 3 (3%)	NA	Peritonitis (27%), patient preference (18%), poor clearance (18%), leak (18%)

Notes: “Never used” was defined as patients with a bAVF who did not transfer to HD (due to kidney transplantation, death, or remained on PD modality), or who transferred to HD with a line. “Stopped working” was defined as patients who did not transfer to HD with a documented bAVF not working or patients who transferred to HD with a line.

Abbreviations: AVF, arteriovenous fistula; bAVF, back-up AVF; HD, hemodialysis; NA, not available; PD, peritoneal dialysis; RPD, remained on PD; Tx, kidney transplantation.

a Mean (standard deviation) per AVF.

b Mean per patient.

**Table 4:** Summary of the proportion of outcomes for bAVF in the PD population

Outcome	All Studies			Excluding Studies Published Before 2000		
	Proportion	95% CI	*I*^2^	Proportion	95% CI	*I*^2^
bAVF stopped working	0.21	0.13-0.31	70.9%	0.20	0.10-0.31	71.8%
bAVF never used	0.69	0.58-0.80	86.2%	0.67	0.54-0.78	83.0%
bAVF never used due to kidney transplant	0.08	0.01-0.20	84.2%	0.07	0.00-0.22	86.5%
bAVF never used due to death	0.11	0.03-0.23	76.7%	0.13	0.04-0.28	82.3%
bAVF never used due to remaining on PD	0.25	0.10-0.44	87.7%	0.26	0.06-0.52	93.9%
Started HD	0.39	0.30-0.49	79.9%	0.41	0.27-0.57	85.7%
Started HD with bAVF	0.30	0.20-0.42	87.1%	0.35	0.23-0.47	81.6%
Started HD with CVC	0.08	0.04-0.15	77.5%	0.07	0.02-0.14	73.5%

Abbreviations: bAVF, back-up arteriovenous fistula; CI, confidence interval; CVC, central venous catheter; HD, hemodialysis; PD, peritoneal dialysis.

Only one study included a breakdown of bAVF complications,^20^ reporting an overall complication rate of 23%. Complications included thrombosis (52%), stenosis (23%), aneurysm formation (10%), and infection (7%). Another study examined time to AVF creation after patient transfer to HD with a CVC,^25^ finding a median time to AVF surgery of 87 days (interquartile range [IQR], 30-179), with a median duration until first use of 149 days (IQR, 104-274) in 18 of 38 patients (47%). Another 2 studies examined the proportion of patients who returned to PD after HD transfer, comparing those with a CVC versus those with a bAVF. One of these studies^25^ reported a return to PD proportion of 19% versus 26%, respectively, while the other study^19^ reported a return to PD proportion of 34% versus 10%, respectively.

### Meta-analysis of Outcomes Comparing bAVF Versus Non-bAVF

**Figure 3:**
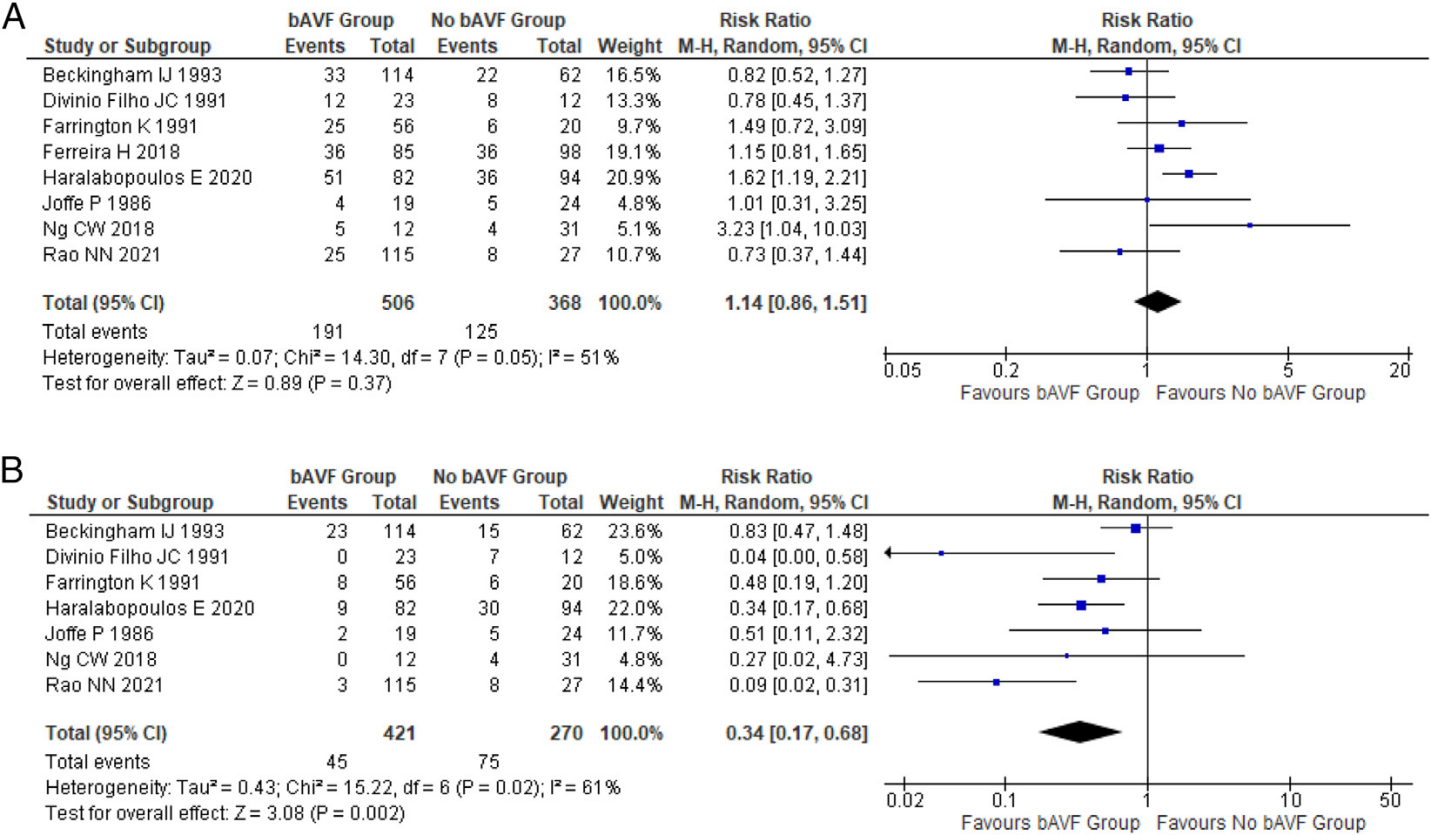
Forest plot showing the risk of (A) commencing hemodialysis and (B) commencing hemodialysis with a central venous catheter between patients with a bAVF and patients with no bAVF. The squares represent the risk ratio and the lines the 95% CIs for the individual studies. The area of each square is proportional to study weight. The diamond and width represent the pooled risk ratios and the 95% CIs, respectively. Abbreviations: bAVF, back-up arteriovenous fistula; CI, confidence interval; M-H, Mantel Haenszel.

Eight studies compared patients with a bAVF to a non-bAVF cohort. A summary of the outcomes in the non-bAVF group is shown in Table S1. There was no difference in the risk of HD transfer between the 2 groups (hazard ratio, 1.14; 95% CI 0.86-1.51; *P* = 0.39; Fig 3A), with moderate heterogeneity across studies (*I*^2^ = 51%).

Seven studies examined the risk of commencing HD with a CVC between the bAVF and non-bAVF cohorts. This showed a significantly lower risk of starting HD with a CVC in favor of the bAVF group (hazard ratio, 0.34; 95% CI, 0.17-0.68; *P* = 0.02; Fig 3B), with moderate heterogeneity across studies (*I*^2^ = 61%).

## Discussion

To our knowledge, this is the first systematic review and meta-analysis to examine bAVF use in the PD population. We demonstrated a significantly lower risk of almost 60% in commencing HD with a CVC in PD patients with a bAVF compared to patients with no bAVF. However, there was a moderate degree of heterogeneity and a large proportion, almost 70%, of bAVFs that were never used. Our study also showed no difference in the proportion of patients who commenced HD between the bAVF and non-bAVF groups.

The main limitation of PD remains the risk of transfer to HD for various reasons. In Australia, almost 50% of PD patients will transfer to HD, with more than half from an infectious cause followed by inadequate dialysis (19%), mechanical failure (18%) and social reasons (11%).^26^ Peritonitis in particular can affect almost half of PD patients^27^^,^^28^ and may result in temporary or permanent transfer to HD. High rates of transfer to HD are further compounded by the low rate of return to PD. Estimates of returning to PD range from only 0.8%-24%, with a lower probability of patients returning to PD the longer they remain on HD.^29^ The high risk of transfer to HD coupled with the low likelihood of returning to PD once HD is initiated is likely what drives individual or unit policy toward creating safe and readily accessible vascular access for acute or chronic HD. Such access would ideally avoid the need to place a CVC for maintenance HD and allows for easy access in case of acute PD disruption.

A bAVF appears to be the most suitable and reasonable choice for such access. Current vascular access and HD guidelines recommend using an AVF for HD due to the lower risk of complications and better clearance than CVCs.^10^^,^^30^, ^31^, ^32^, ^33^ Compared to AVF, CVCs significantly increase the risk of infection related hospital admission,^34^^,^^35^ central vein stenosis,^35^ and poorer blood flow and clearance.^35^ Outcomes of AVF are also poorer in patients with a previous CVC insertion,^36^ with one Australian study showing an 80% increased risk of AVFs failing to mature in patients with a previous CVC and an increased risk of interventions to assist maturation.^37^ In our analysis, the creation of a bAVF showed a clear reduction of almost 70% in the risk of starting HD with a CVC, which would support the practice of its use.

Despite the clear advantages of an AVF as a vascular access for HD, there are limitations that are also applicable to PD patients with a bAVF. The utility of an AVF depends on its successful maturation and function, and primary failure of AVFs remains a barrier. In the HD population, the rate of maturation failure is high, ranging from 30%-60%.^38^^,^^39^ Patency rates are low at 65% in 1 year^40^ and 66% in 2 years.^41^ Up to 44% of AVFs will require an intervention to facilitate maturation, with the mean number of procedures needed to achieve maturation ranging from 2.9-3.9.^42^ Our study supports these trends in the PD population as well, with our meta-proportional analysis confirming 20% of bAVFs stopped working and 70% were never used. This number is likely underestimated because none of the studies reported whether the bAVFs were subject to routine surveillance, and it is possible that failure of bAVFs to mature may only have been noted at the time access was needed for HD.

These limitations understandably discourage the universal creation of bAVFs in all PD patients. The decision to create a bAVF should balance the concern of transfer to HD and starting HD with a CVC against the risks of bAVF creation. These risks include failure to mature, high proportion of non-use, and requirement for repeated procedures, in addition to the financial costs associated with creation, maintenance, and active surveillance. Careful patient selection is a better approach taking these factors into account and including patient preference after advising and counseling on the advantages of a bAVF and possible complications. It may be useful to consider factors associated with risk of transfer to HD when assessing a PD patient’s need for a bAVF. Patients at risk of transfer to HD include those from smaller dialysis centers (<20 PD patients)^6^^,^^43^ and those with older age or comorbid conditions such as cardiovascular disease and diabetes.^44^ Finally, it is important to note that in our study, we did not find a difference in the risk of starting HD between the bAVF and non-bAVF group, highlighting the probable lack of thorough patient selection, which may be needed to make the policy of creating bAVFs valid and justified.

To date there has been no formal definition for what constitutes a bAVF in the PD population, with our systematic review showing variations in the definition of bAVF in different studies. This includes an AVF used for HD before patient transfer to PD,^11^^,^^17^^,^^24^ having a bAVF created in all incident PD patients (unless not surgically suitable),^12^ patients who had an AVF created after PD commencement in anticipation of starting HD,^23^^,^^25^ or high-risk PD patients who were specifically selected for bAVF creation due to concerns about transfer to HD.^12^^,^^21^^,^^25^ A definition of a bAVF should be proposed for better standardization, reporting, and research clarity. It may be best for definitions to be proposed by a consensus working group, such as one dedicated to creating guidelines, to capture the diverse range of opinions, including those of consumers. Based on our analysis, we suggest the following recommendations to be considered when defining a bAVF with the aim of achieving uniformity and a clear direction for future research: a bAVF is an arteriovenous access created or present and receiving ongoing maintenance and surveillance in a PD patient aimed at being used in the event of transfer to HD to minimize the risk of starting HD with a CVC. bAVFs should be divided and grouped as (1) existing AVF used during HD before PD transfer; (2) created specifically pre-PD start or up to 6 months after PD commencement; (3) created >6 months after PD commencement (because after 6 months, it is likely that most AVFs are created in anticipation of transfer to HD). We anticipate that each group is likely to have a different success rate, use rate, and reasons for creation. Such definitions will also allow us to appreciate variations in practice, which may be due to resources such as timely vascular surgery access, presence of a dedicated vascular access nurse, and unit policies.

It is not clear how common the practice of creating a bAVF is or the proportion of centers that use this back-up. A survey of units in the United Kingdom in the early 1990s showed this practice was present in up to 42% of the 70 units surveyed.^19^ Although only a limited number of publications have examined bAVFs, our review identified centers in Europe, North America, South America and Australia, indicating this practice may be more widespread than appreciated but underreported.

In our analyses, heterogeneity was high for the proportion of bAVFs never used and inpatients with a bAVF who started HD with a CVC or a bAVF. However, we found moderate heterogeneity in the meta-analysis comparing the risk of starting HD with a CVC between the bAVF and non-bAVF group. This heterogeneity make our results difficult to interpret and generalize for several reasons. First, the definition of what constitutes a bAVF was not uniform across the studies. Second, studies were included from as far back as 1980 until 2020, making it likely that changes and improvements in treatment modalities, management of PD patients, and AVF creation techniques result in different era effects. However, we did attempt to account for this by conducting a sensitivity analysis and including only studies published after 2000. Third, the reason for why the bAVF was created or the patient selection process was not clearly stated in most studies. Finally, outcomes were not clearly and uniformly documented consistently across the studies.

To our knowledge, this is the only analysis featuring a meta-proportional analysis and meta-analysis examination of bAVFs in the PD population. We used a comprehensive search strategy, including the gray literature, to identify abstracts, letters, short reports, and published studies to pool a large number of patients across countries and services, allowing for greater generalizability.

Our analysis also has several limitations, with some inherent to the study design of the included articles. There were no randomized controlled trials, and none of the included studies were prospective, limiting our analysis to retrospective data, which increases the risk of selection bias and data accuracy. In studies with 2 groups, bAVF and non-bAVF, there was no analysis or consideration for matching or balancing the groups. Outcomes were limited to bAVF use, failure, and starting HD between the cohorts and groups with no study examining survival, cardiovascular events or infection risk, patient satisfaction, or cost analysis, which would have been more relevant to policy makers and guideline working groups. As discussed, substantial heterogeneity existed among the studies, which was unsurprising given the retrospective, mostly single-centered observational nature of the included studies. For some outcome measures, the number of included studies was small and therefore, the results need to be interpreted with caution. This was especially the case for reasons a bAVF was never used (transplantation, death, or remained on PD), for which only 5 studies included the relevant data. Despite these limitations, we still collected 11 studies in total for a meta-proportional analysis and 8 studies for a meta-analysis on the outcome of b-AVFs in the PD population. This was a sufficient prerequisite to perform an analysis.

In conclusion, our analysis showed that 20% of bAVFs in the PD population failed to mature and that a large proportion were never used. Despite this, the risk of commencing HD using a CVC was lower in the presence of a bAVF. Our data suggests that the practice of using a bAVF may be justified; however, caution may be needed in patient selection. Our analysis can help units establish protocols, assess risk benefits for adopting a bAVF policy, and better inform patients. Future research should consider a randomized controlled trial to examine the utility of bAVFs in the PD population. Outcomes will need to be better defined in future studies to include bAVF maturity and use, survival between the 2 groups (bAVF and non-bAVF), rates of cardiovascular events, and infection risk, as well as cost comparisons and measures of patient satisfaction.

## Supplementary Material

Supplementary File (PDF) Figures S1-S8; Item S1; Table S1.
