# Supplementary material for: Back-up Arteriovenous Fistulas in Peritoneal Dialysis Patients: A Systematic Review and Meta-analysis
Source: Kidney Med. 2024 Sep 19;6(11):100904. doi: 10.1016/j.xkme.2024.100904 (PMC11543892; doi:10.1016/j.xkme.2024.100904)
Supplement: Supplementary File (PDF) — Figures S1-S8; Item S1; Table S1. [file mmc1.docx]

Item S1: Search strategy in Medline and Embase

MEDLINE(R) including Daily update <1996-current>

1 exp Peritoneal Dialysis/
2 exp Arteriovenous Fistula/
3 exp Vascular Fistula/
4 exp Arterio-Arterial Fistula/
5 exp Arteriovenous Shunt, Surgical/
6 2 or 3 or 4 or 5
7 peritoneal dialysis.tw.
8 fistula.tw.
9 (vascular access or venous access).tw.
10 (dialysis access or haemodialysis access).tw.
11 1 or 7
12 6 or 8 or 9 or 10
13 11 and 12

Embase <1996 to 2021 November 19>

1 exp peritoneal dialysis/
2 exp arteriovenous fistula/
3 exp blood vessel fistula/
4 exp arteriovenous shunt/
5 2 or 3 or 4
6 peritoneal dialysis.tw.
7 fistula.tw.
8 AVF.tw.
9 (vascular access or venous access).tw.
10 (dialysis access or haemodialysis access).tw.
11 1 or 6
12 5 or 7 or 8 or 9 or 10
13 11 and 12


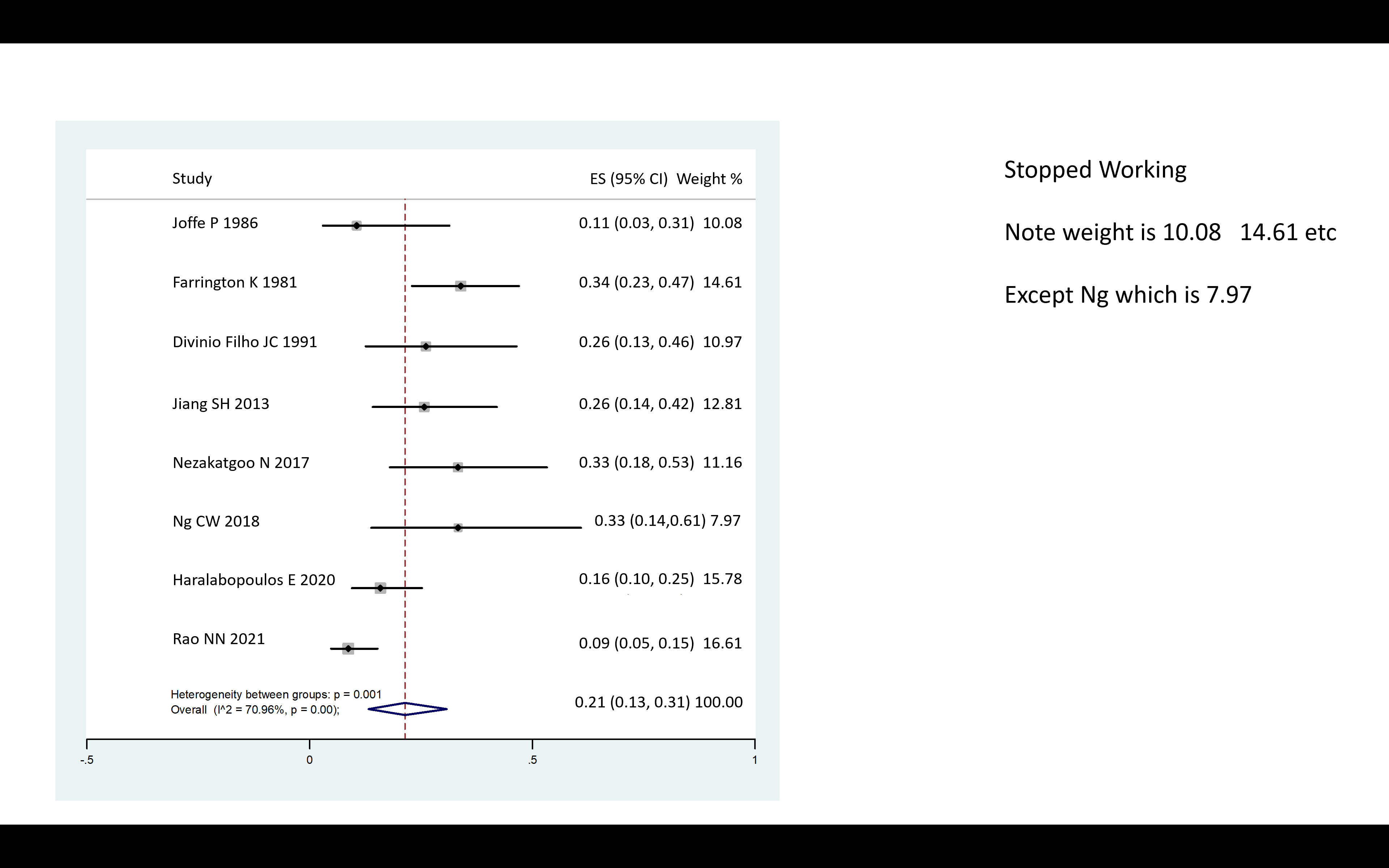


Figure S1: Overall bAVF that stopped working forest plot


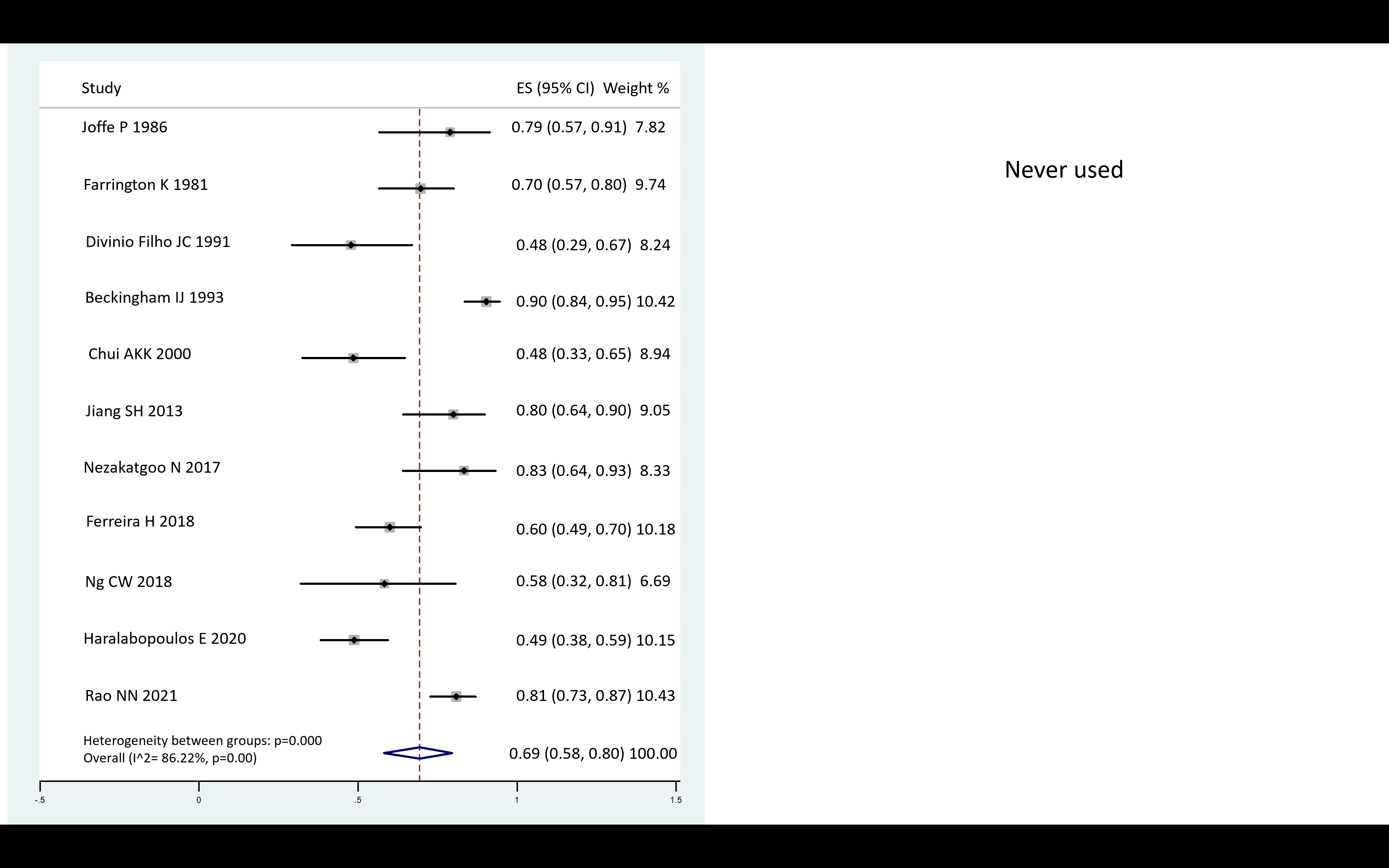


Figure S2: Overall bAVF that were never used forest plot


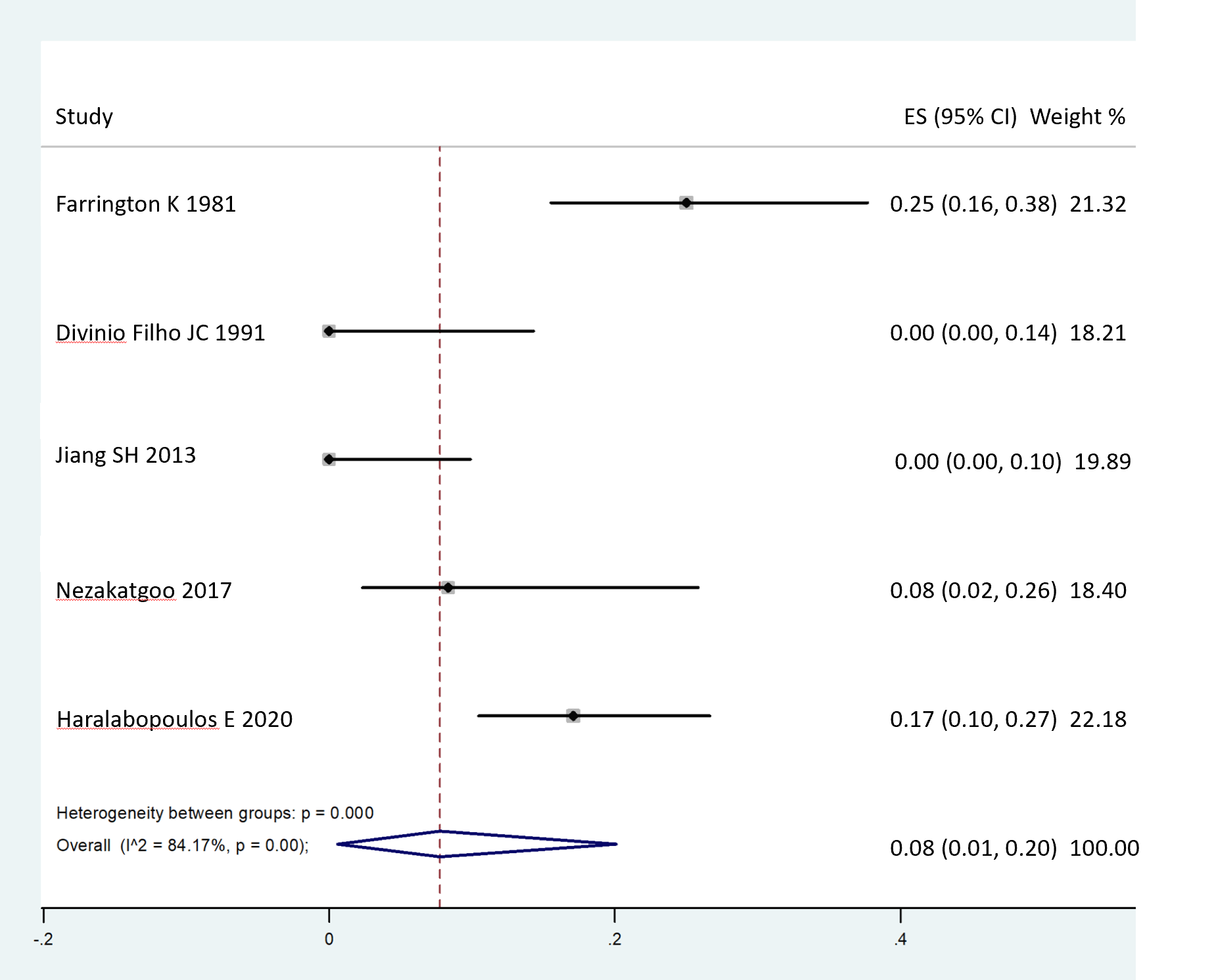


Figure S3: Overall bAVF that were never used due to kidney transplantation


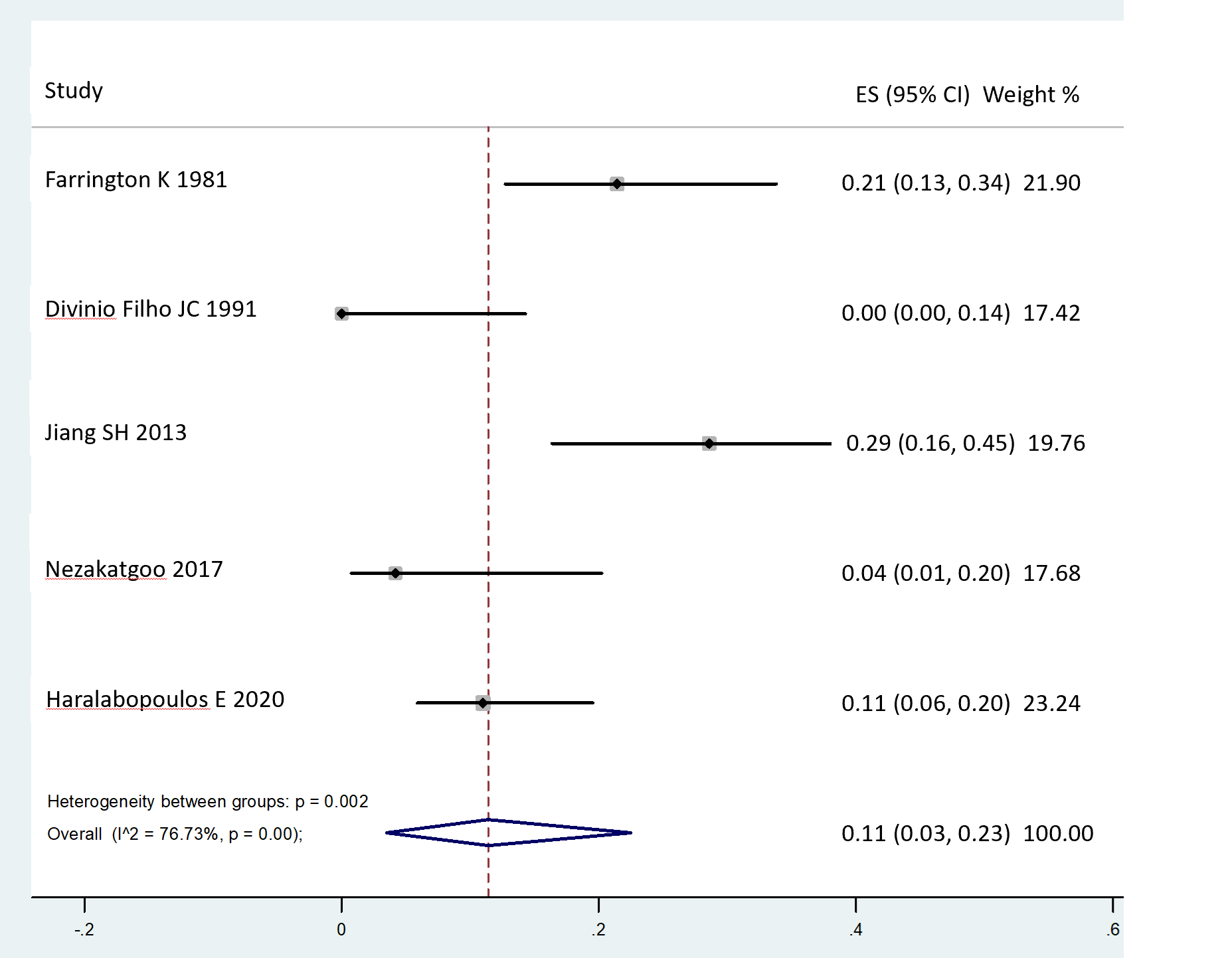


Figure S4: Overall bAVF that were never used due to death


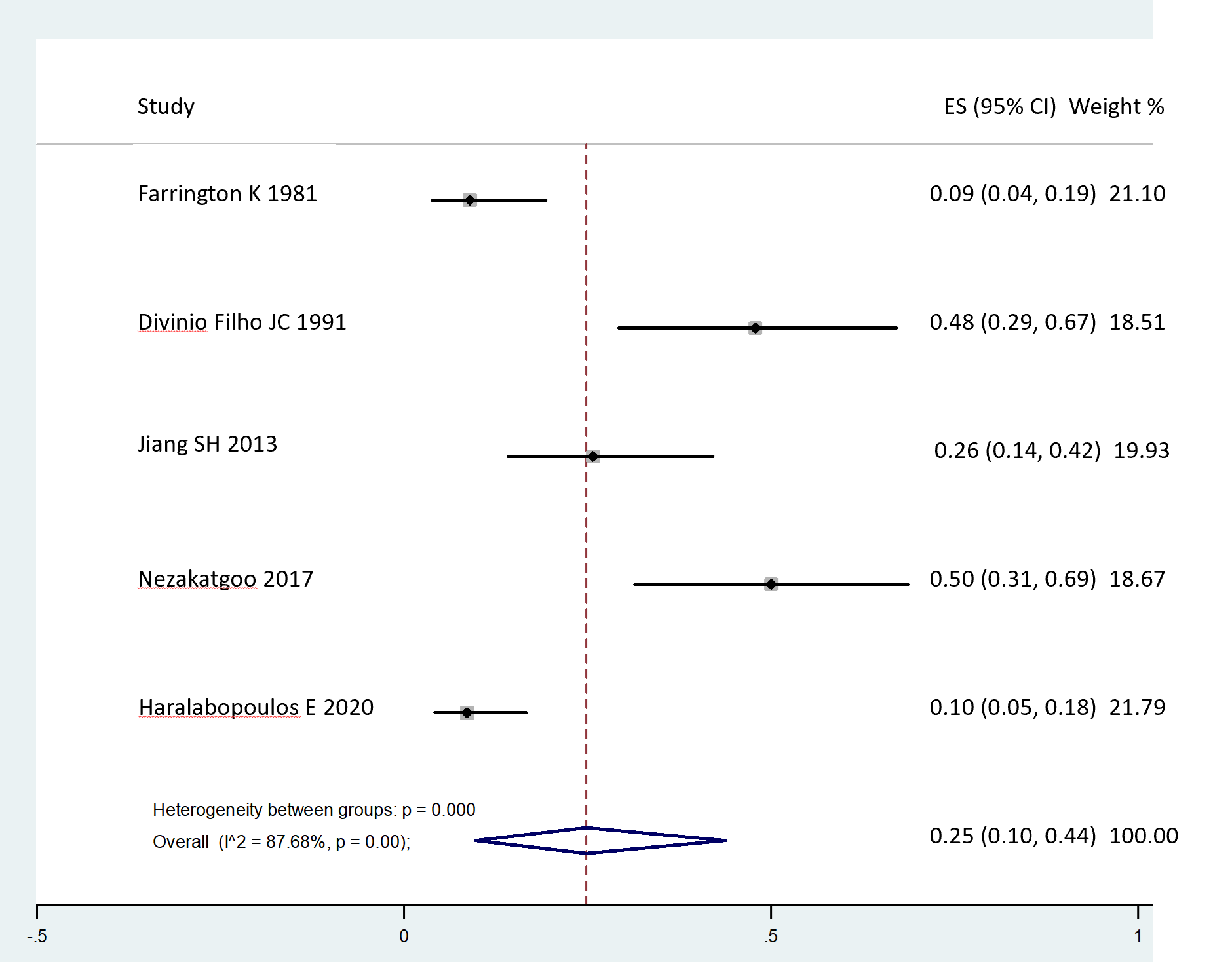


Figure S5: Overall bAVF that were never used due to remaining on PD


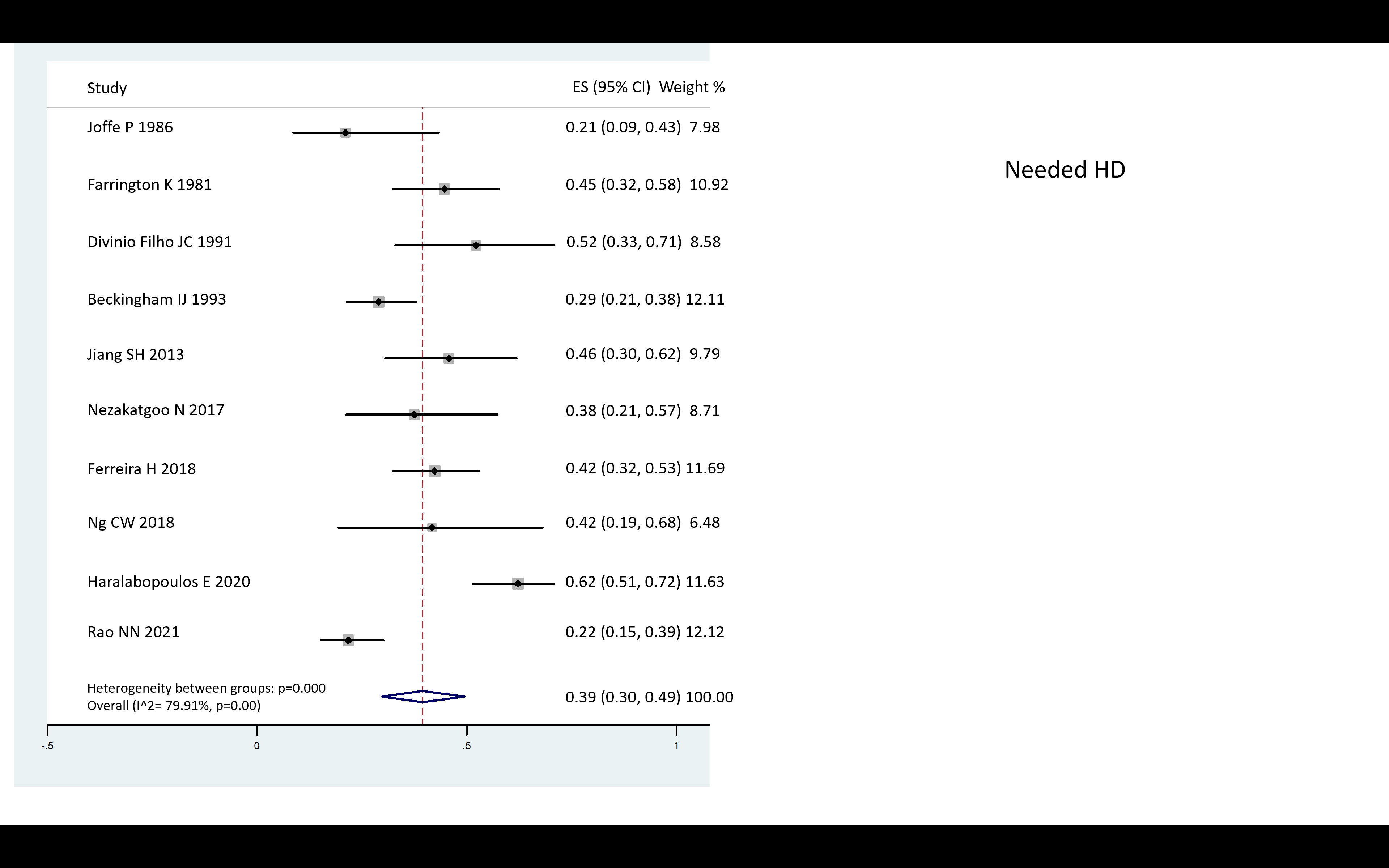


Figure S6: Overall patients with a bAVF who commenced HD forest plot


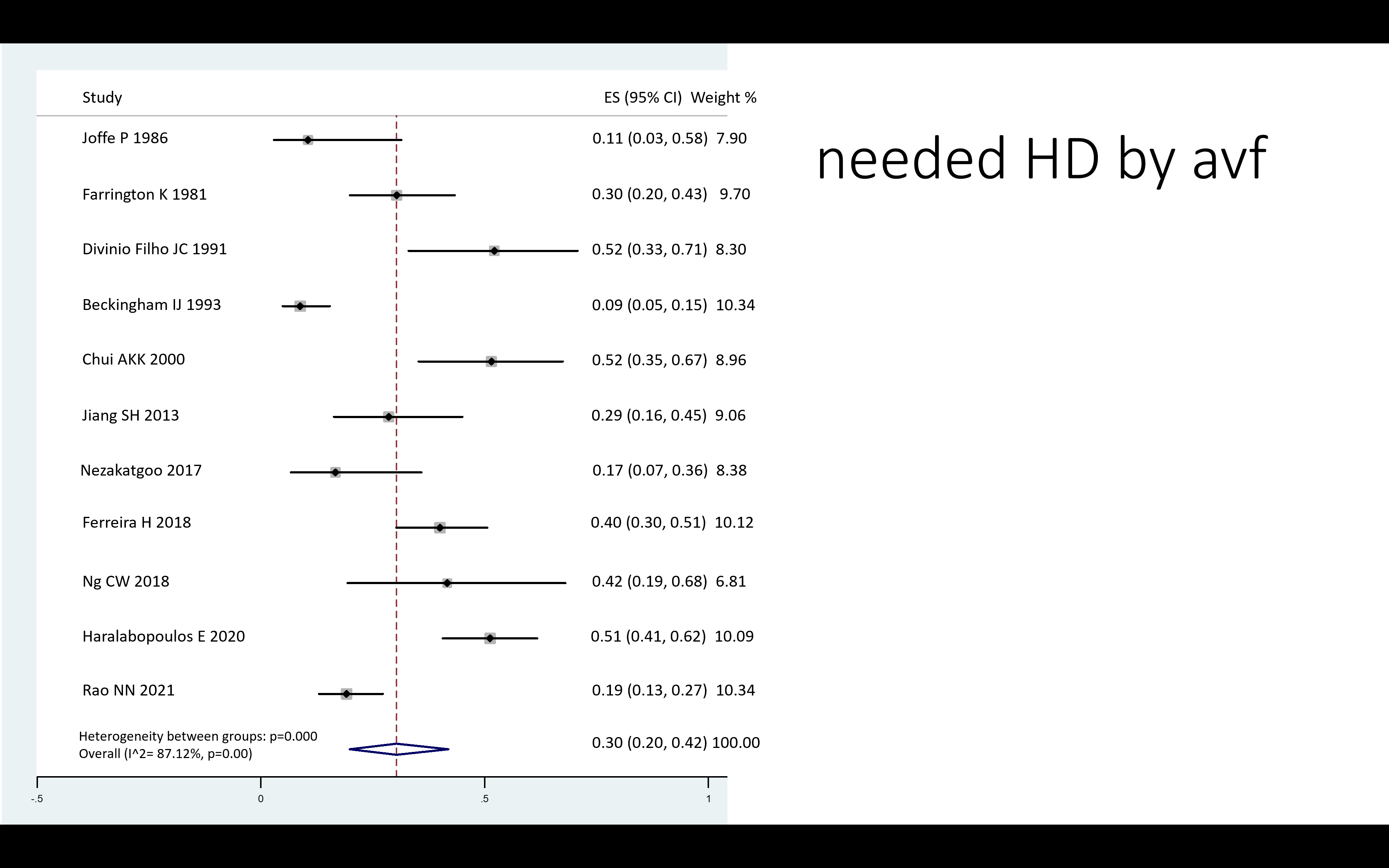


Figure S7: Overall patients with a bAVF who commenced HD with a bAVF forest plot


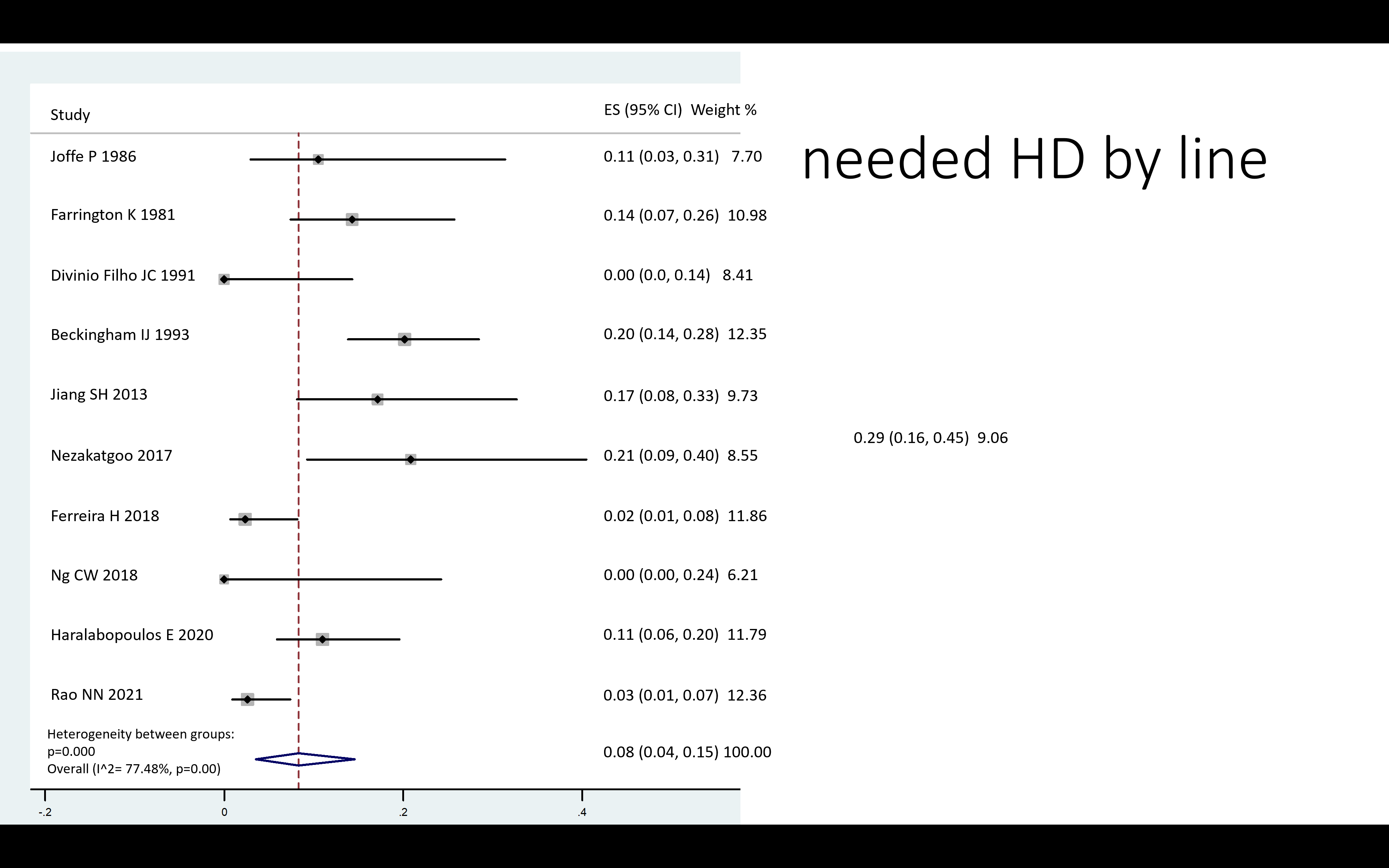


Figure S8: Overall patients with a bAVF who commenced HD with a CVC forest plot

| **Table S1: Outcomes of included studies with information on non-bAVF cohorts** | | | | | | | |  |  |
| --- | --- | --- | --- | --- | --- | --- | --- | --- | --- |
| **Author (Year)** | | **Total Number of none bAVF** | **Requiring HD** | | **Requiring HD through line** | **Requiring HD through AVF** | |  |  |
| Joffe P (1986) | | 24 | 5 | | 5 | 0 | |  |  |
| Farrington K (1991) | | 20 | 6 | | 6 | 0 | |  |  |
| Divino Fiho JC (1991) | | 12 | 8 | | 7 | 1 | |  |  |
| Beckingham IJ (1993) | | 62 | 22 | | 15 | NA | |  |  |
| Ferreira H (2018) | | 98 | 36 | | NA | NA | |  |  |
| Ng CW (2018) | | 31 | 4 | | 4 | 0 | |  |  |
| Haralabopoulos E (2020) | | 94 | 36 | | 30 | 6 | |  |  |
| Rao NN (2021) | | 27 | 8 | | 8 | 0 | |  |  |
|  | | | | | | | |  |  |
|  |  | | |  | | |  | |  |
